# Levonorgestrel at emergency contraception dose has no effect on ciliary beat frequency and muscular contraction of the human fallopian tube: an in vitro experimental study

**DOI:** 10.1186/s12958-024-01315-6

**Published:** 2024-11-19

**Authors:** Raymond Hang Wun Li, Su-Bin Liao, William Shu Biu Yeung, Ernest Hung Yu Ng, Wai Sum O, Pak Chung Ho

**Affiliations:** Department of Obstetrics and Gynaecology, School of Clinical Medicine, Li Ka Shing Faculty of Medicine, The University of Hong Kong, 102 Pokfulam Road, Pok Fu Lam, Hong Kong

**Keywords:** Levonorgestrel, Emergency contraception, Fallopian tube, Ciliary beat frequency, Muscular contraction

## Abstract

**Background:**

Levonorgestrel (LNG) acts as an emergency contraceptive mainly by inhibiting or postponing ovulation. We carried out this study to investigate whether LNG at concentration relevant for emergency contraception (EC) affect ciliary beat frequency and muscular contraction of the human Fallopian tube, which might contribute additional actions for EC.

**Methods:**

This was an in vitro experimental study on human Fallopian tube tissue collected from ten women undergoing hysterectomy. The tubal explants were cultured in vitro, primed with oestradiol and progesterone at concentrations resembling the physiological early luteal phase, and treated with LNG at 0, 1, 10 and 100 ng/ml concentrations. Ciliary beat frequency was measured from the tubal epithelial strips, and the basal tone, amplitude and frequency of contractions were recorded from longitudinal smooth muscle strips. These parameters at different LNG concentrations were compared against the control (LNG 0 ng/ml).

**Results:**

Treatment of tubal tissue strips with LNG at all concentrations studied did not significantly alter the ciliary beat frequency nor basal muscle tone (*p* > 0.05 for both) compared with control. Significant reduction in the amplitude and frequency of tubal muscular contractions was shown after treatment with LNG only at 100 ng/ml (*p* < 0.05 for both) but not lower concentrations (*p* > 0.05) compared with control.

**Conclusion:**

LNG did not significantly inhibit ciliary beat frequency and muscular contraction of the human Fallopian tube at the doses used for EC, suggesting that the Fallopian tube is unlikely a target for the EC action of LNG.

## Background

Emergency contraception (EC) serves as an important back-up contraceptive method in cases of unprotected intercourse or failure of a regular contraceptive method. The EC pill containing levonorgestrel (LNG) 1.5 mg taken within 72 h after unprotected intercourse is currently a recommended oral EC method used worldwide. It is recognised that postponement or inhibition of ovulation is the main mechanism of its EC action [1, 2]. There are limited data on whether LNG has effects on the functions of the human Fallopian tube, which might contribute additionally to its contraceptive actions. Previous biomedical studies suggested that LNG at supra-pharmacological concentrations could reduce ciliary beat frequency (CBF) and/or muscular contraction frequencies in human Fallopian tube explants [3–5], but these may not be extrapolated to LNG at pharmacological doses used for EC. On the other hand, our previous study suggested that both ulipristal acetate and mifepristone, another two recommended agents for EC, could inhibit CBF and muscular contractility in human Fallopian tube tissue [6].

The current study aimed at examining the effect of LNG at pharmacological doses on functions of human Fallopian tube, namely CBF and muscular contractions, in an optimised experimental condition.

## Methods

This was an in-vitro experimental study using the isthmic part of human Fallopian tubes obtained from ten women who underwent hysterectomy in Queen Mary Hospital, Hong Kong, for benign gynaecological conditions not involving the Fallopian tubes. Ethics approval was obtained from the Institutional Review Board, The University of Hong Kong / Hospital Authority Hong Kong West Cluster. These women gave written informed consent prior to the operation.

The tubal epithelium and longitudinal smooth muscle fibers of the collected tubes were dissected out into small pieces and rinsed for multiple times in ice-cold Dulbecco’s modified Eagle’s medium/F12 medium supplemented with 10% fetal bovine serum (FBS) (Invitrogen, Carlsbad, CA, USA) to remove visible blood. This was followed by overnight incubation with oestradiol 100 pmol/L and progesterone 10 nmol/L; these hormones were added to prime and condition the tubal tissue to a hormonal environment resembling the physiological early luteal phase.

A portion of these tissue strips from six of the recruited women (at least 9 strips from each) were then treated with LNG at graded concentrations of 0, 1, 10 and 100 ng/ml. LNG at 10 ng/ml approximately corresponded to its peak serum concentration after a pharmacological oral dose of 1.5 mg [7]. After the above treatments, CBF of the treated tubal epithelium strips was measured using a photometric method as previously described [8, 9]. CBF was captured by a photo-multiplier and translated digitally.

Strips of tubal smooth muscle from all ten women were connected to a force transducer coupled to a graphic recorder, while bathed in Kreb’s solution. After equilibration for 30 min, LNG was added at 1, 10 and 100 ng/ml to the medium at 2 min intervals, and the contraction tracing was recorded graphically. The mean basal tone, contraction frequency and amplitude were determined digitally. Details of the experimental methodology followed that of our previously reported study on ulipristal and mifepristone [6].

CBF recorded after LNG treatment at the different concentrations were compared by Kruskal-Wallis test with Dunn’s multiple comparison post-hoc analysis, and measures of contractility were compared using Friedman’s test with Dunn’s multiple comparison post-hoc analysis. Statistical analyses were performed by the GraphPad Prism 10 software (GraphPad, San Diego, CA USA). A p-value of less than 0.05 was considered statistically significant.

## Results

**Fig. 1:**
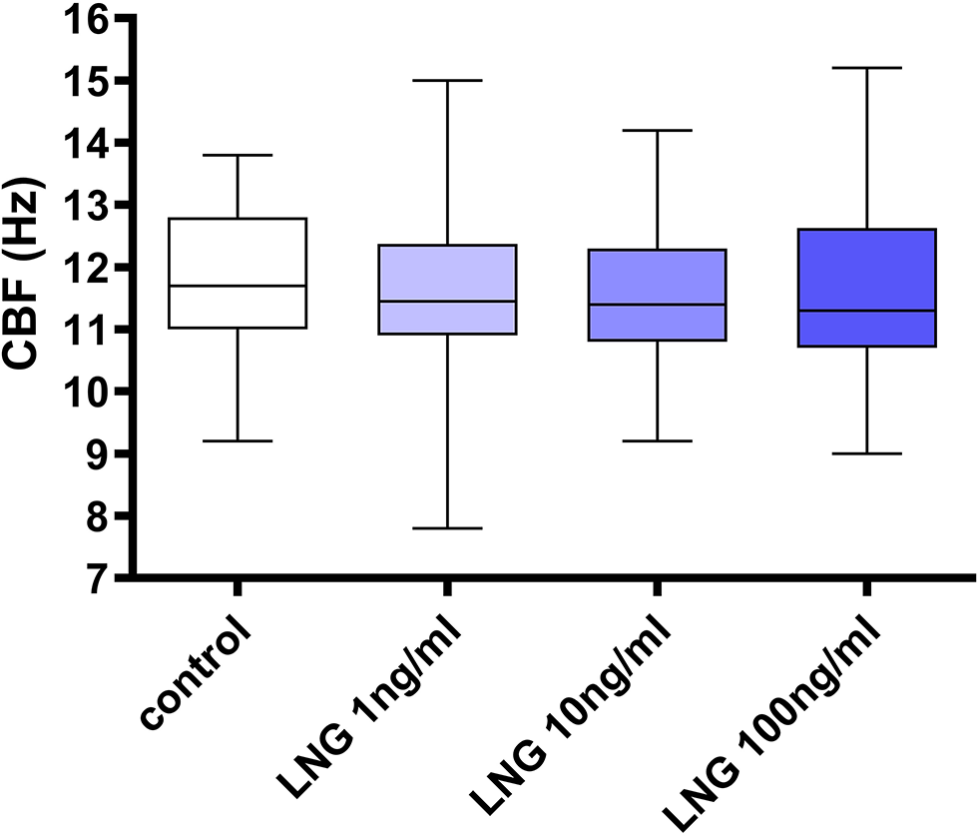
Effect of levonorgestrel (LNG) treatment on ciliary beat frequency (CBF) of human Fallopian tube

**Fig. 2:**
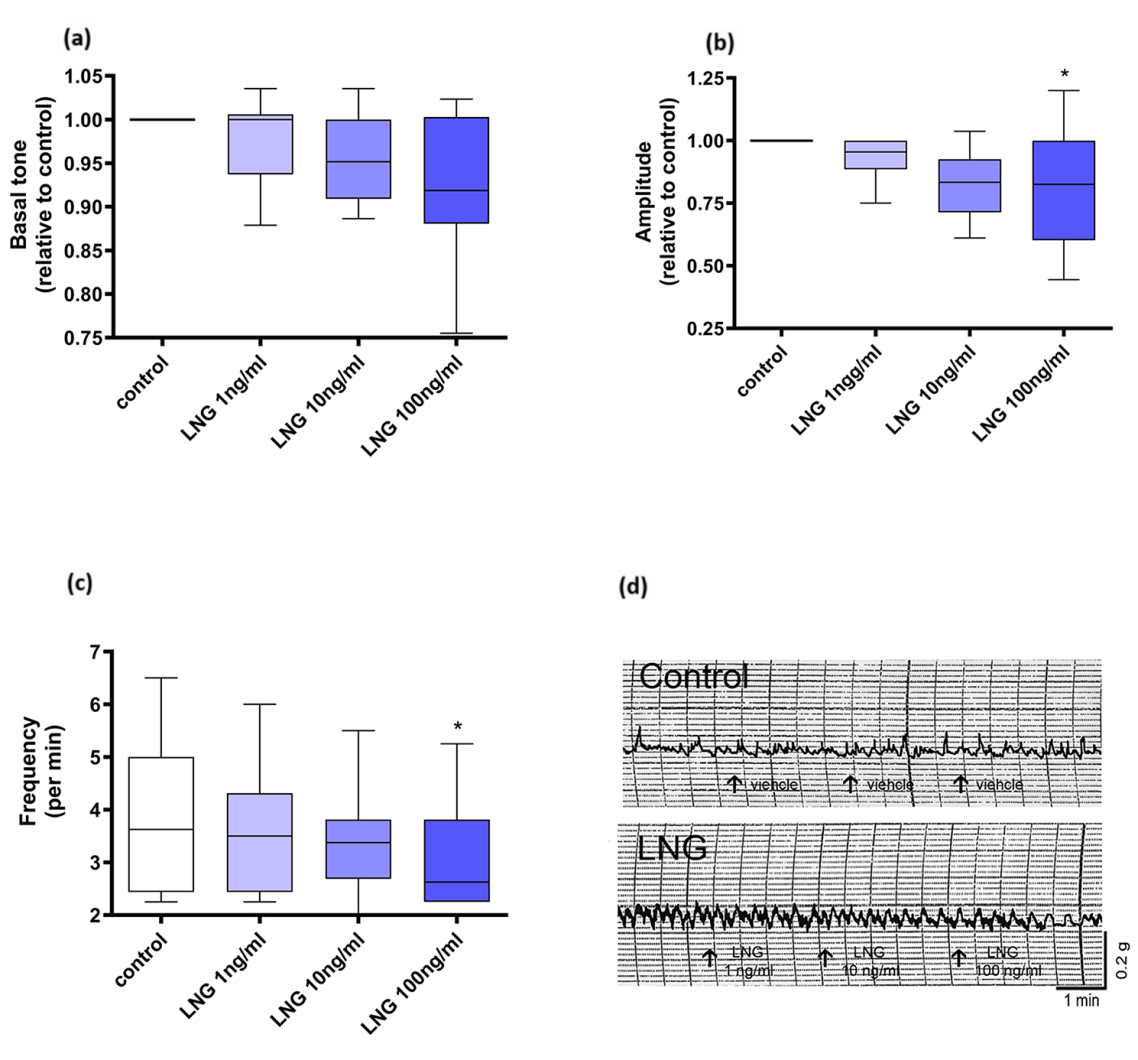
Effect of levonorgestrel (LNG) treatment on smooth muscle contraction parameters including (**a**) basal tone, (**b**) amplitude and (**c** frequency of human Fallopian tube. A representative tracing of the muscular contraction recording is shown in Panel (**d**). (*p < 0.05)

There was no significant change in CBF after treatment with LNG at all concentrations studied compared with control (*p* > 0.05) (Fig. 1). We have reviewed the between-subject and within-subject variations and no significant difference was demonstrated (Kruskal-Wallis test). Regarding muscular contractility, there was no significant change in basal tone after treatment with LNG at all concentrations studied compared with control (*p* > 0.05) (Fig. 2a), while significant reduction in amplitude (*p* < 0.05) (Fig. 2b) and frequency (*p* < 0.05) (Fig. 2c) was noted after treatment with LNG only at 100 ng/ml but not lower doses (*p* > 0.05). A representative tracing of the muscular contraction recording is shown in Fig. 2d.

## Discussion

Our results demonstrated that LNG at concentrations relevant for EC, i.e. 10 ng/ml or below, did not have significant effect on CBF and smooth muscle contraction of the human Fallopian tube, although a significant suppression of contraction amplitude and frequency was demonstrated after treatment with LNG at a supra-pharmacological concentration of 100 ng/ml.

A previous report showed that LNG at supra-pharmacological (1 µM or above, i.e. over 312 ng/ml), but not 0.1µM or lower concentrations, significantly reduced CBF in human Fallopian tube explants [5]. Another study [3] showed that LNG at both sub-pharmacological (0.02 µM, i.e. 6.25 ng/ml) and supra-pharmacological doses (0.2 µM, i.e. 62.5 ng/ml) significantly reduced the muscular contraction frequency and area under the curve, which was discrepant with our findings. In these two studies, the tubal explants were not primed with a standard oestradiol and progesterone condition resembling the early luteal phase milieu as what we did; the difference between the control and treatment groups might hence be more exaggerated as progesterone is expected to exert a quiescent effect on the Fallopian tube. A further study [4] investigated human Fallopian tube explants collected at the secretory phase and found that in vitro treatment with LNG significantly reduced CBF at concentrations of 0.5 and 5 µM (i.e. 156 and 1560 ng/ml), which again were well above the pharmacological dose. Our study attempted to fill the above gaps.

Theoretically, a reduction in CBF and/or muscular contraction of the Fallopian tube may result in delayed transport of the early embryo to the endometrial cavity and hence dys-synchronisation with the implantation window, thereby reducing the probability of conception. This may contribute an additional mechanism towards a contraceptive effect. In real life, it is not certain how much it can contribute to an enhancement of efficacy in the EC setting. On the other hand, there may be a theoretical concern that if LNG-EC could retard Fallopian tube activities, this may result in an increased propensity of embryo retention and hence risk of ectopic pregnancy. However, a systematic review did not show evidence of increased risk of ectopic pregnancy after intake of LNG-EC [10]. Nonetheless, the negative effect that we observe with LNG administered at concentrations relevant to the EC context probably rules out a significant impact of LNG on the Fallopian tube clinically.

We consider a few strengths of our study. First, we treated the Fallopian tube tissue with LNG at graded concentrations covering that corresponding to the peak serum level after a pharmacological EC dose. Second, we conditioned the tissue with a standard concentration of estradiol and progesterone to mimic the early luteal phase, which minimised confounding effects of the samples being collected from women at different phases of their menstrual cycles. Third, we employed the same experimental methods which we adopted in previous studies that successfully demonstrated the effect of in vitro drug treatments with mifepristone and ulipristal acetate, which could serve as a historical control. Although the sample size of our study was limited, it was comparable to that of our previous study which showed significant effects of ulipristal and mifepristone at pharmacological doses. As for most in vitro biomedical studies, there was no a priori sample size calculation. A retrospective sample size estimation was performed for assumed power of 80% and type I error of 0.05. For ciliary beat frequency, the standard deviation of our cohort was 1.1. A minimum sample size of 21 tissue strips is adequate to demonstrate a difference of 1. For muscular tone, the standard deviation of our cohort at the lowest LNG concentration was 0.05; a minimum sample size of 5 tissue strips is required to demonstrate a difference of 0.1 (in arbitrary units) by repeated sample analysis. Hence, the size of our samples studied should be considered adequate.

## Conclusions

Our study found that LNG did not inhibit ciliary beat and muscular contraction of the human Fallopian tube at the pharmacological dose used for EC, suggesting that the Fallopian tube is unlikely a target for the EC action of LNG.

## Data Availability

The datasets used and/or analysed during the current study are available from the corresponding author on reasonable request.
